# Supplementary material for: Resolving the full spectrum of human genome variation using Linked-Reads
Source: Genome Res. 2019 Apr;29(4):635–45. doi: 10.1101/gr.234443.118 (PMC6442396; doi:10.1101/gr.234443.118)
Supplement: Supplemental Material [file supp_gr.234443.118_Supplemental_longranger-2.2.zip › longranger-2.2.tar.bz2!/lr2.2-public/lib/go/src/code.google.com/vitess/docs/user-guide/server-configuration.html]

Vitess / Server Configuration


Toggle navigation

- Overview
- Guides
- Reference
- Blog
- GitHub

- #### Overview

  - What is Vitess
  - Scaling MySQL with Vitess
  - Key Concepts
- #### Getting Started

  - Run Vitess on Kubernetes
    - Custom Docker Build
  - Run Vitess Locally
- #### User Guide

  - Introduction- Client Libraries- Backing Up Data- Reparenting
        - Schema Management
        - VSchema Guide
        - Schema Swap (Tutorial)
        - Sharding
          - Horizontal Sharding (Tutorial, automated)
          - Horizontal Sharding (Tutorial, manual)
          - Sharding in Kubernetes (Tutorial, automated)
          - Sharding in Kubernetes (Tutorial, manual)
        - Vitess Sequences
        - MySQL Server Protocol
        - Vitess and Replication
        - Update Stream
        - Row Based Replication
        - Topology Service
        - Transport Security Model
        - Launching
          - Scalability Philosophy
          - Production Planning
          - Server Configuration
          - 2PC Guide
          - Troubleshooting
        - Upgrading
- #### Reference Guides

  - Vitess API- vtctl Commands
- #### Other Resources

  - Presentations- Blog- Roadmap
- #### Contributing

  - Contributing to Vitess- GitHub Workflow- Code Reviews
- GitHub

# Server Configuration

- #### Overview

  - What is Vitess
  - Scaling MySQL with Vitess
  - Key Concepts
- #### Getting Started

  - Run Vitess on Kubernetes
    - Custom Docker Build
  - Run Vitess Locally
- #### User Guide

  - Introduction- Client Libraries- Backing Up Data- Reparenting
        - Schema Management
        - VSchema Guide
        - Schema Swap (Tutorial)
        - Sharding
          - Horizontal Sharding (Tutorial, automated)
          - Horizontal Sharding (Tutorial, manual)
          - Sharding in Kubernetes (Tutorial, automated)
          - Sharding in Kubernetes (Tutorial, manual)
        - Vitess Sequences
        - MySQL Server Protocol
        - Vitess and Replication
        - Update Stream
        - Row Based Replication
        - Topology Service
        - Transport Security Model
        - Launching
          - Scalability Philosophy
          - Production Planning
          - Server Configuration
          - 2PC Guide
          - Troubleshooting
        - Upgrading
- #### Reference Guides

  - Vitess API- vtctl Commands
- #### Other Resources

  - Presentations- Blog- Roadmap
- #### Contributing

  - Contributing to Vitess- GitHub Workflow- Code Reviews

## MySQL

Vitess has some requirements on how MySQL should be configured. These will be detailed below.

As a reminder, semi-sync replication is highly recommended. It offers a much better durability story than relying on a disk. This will also let you relax the disk-based durability settings.

### Versions

MySQL versions supported are: MariaDB 10.0, MySQL 5.6 and MySQL 5.7. A number of custom versions based on these exist (Percona, …), Vitess most likely supports them if the version they are based on is supported.

### Config files

#### my.cnf

The main `my.cnf` file is generated by
mysqlctl init
based primarily on
$VTROOT/config/mycnf/default.cnf.
Additional files will be appended to the generated `my.cnf` as specified in
a colon-separated list of absolute paths in the `EXTRA_MY_CNF` environment
variable. For example, this is typically used to include flavor-specific
config files.

To customize the `my.cnf`, you can either add overrides in an additional
`EXTRA_MY_CNF` file, or modify the files in `$VTROOT/config/mycnf` before
distributing to your servers. In Kubernetes, you can use a
ConfigMap to overwrite
the entire `$VTROOT/config/mycnf` directory with your custom versions,
rather than baking them into a custom container image.

#### init\_db.sql

When a new instance is initialized with `mysqlctl init` (as opposed to
restarting in a previously initialized data dir with `mysqlctl start`),
the init\_db.sql
file is applied to the server immediatley after executing `mysql_install_db`.
By default, this file contains the equivalent of running
mysql\_secure\_installation,
as well as the necessary tables and grants for Vitess.

If you are running Vitess on top of an existing MySQL instance,
rather than using mysqlctl, you can use this file as a sample of what
grants need to be applied to enable Vitess.

Note that changes to this file will not be reflected in shards that have
already been initialized and had at least one backup taken.
New instances in such shards will automatically restore the latest backup
upon vttablet startup, overwriting the data dir created by mysqlctl.

### Statement-based replication (SBR)

Vitess relies on adding comments to DMLs, which are later parsed on the other end of replication for various post-processing work. The critical ones are:

- Redirect DMLs to the correct shard during resharding workflow.
- Identify which rows have changed for notifying downstream services that wish to subscribe to changes in vitess.
- Workflows that allow you to apply schema changes to replicas first, and rotate the masters, which improves uptime.

In order to achieve this, Vitess also rewrites all your DMLs to be primary-key based. In a way, this also makes statement based replication almost as efficient as row-based replication (RBR). So, there should be no major loss of performance if you switched to SBR in Vitess.

RBR will eventually be supported by Vitess.

### Data types

Vitess supports data types at the MySQL 5.5 level. The newer data types like spatial or JSON are not supported yet. Additionally, the TIMESTAMP data type should not be used in a primary key or sharding column. Otherwise, Vitess cannot predict those values correctly and this may result in data corruption.

### No side effects

Vitess cannot guarantee data consistency if the schema contains constructs with side effects. These are triggers, stored procedures and foreign keys. This is because the resharding workflow and update stream cannot correctly detect what has changed by looking at a statement.

This rule is not strictly enforced. You are allowed to add these things, but at your own risk. As long as you’ve ensured that a certain side-effect will not break Vitess, you can add it to the schema.

Similar guidelines should be used when deciding to bypass Vitess to send statements directly to MySQL.

Vitess also requires you to turn on STRICT\_TRANS\_TABLES mode. Otherwise, it cannot accurately predict what will be written to the database.

It’s safe to apply backward compatible DDLs directly to MySQL. VTTablets can be configured to periodically check the schema for changes.

There is also work in progress to actively watch the binlog for schema changes. This will likely happen around release 2.1.

### Autocommit

MySQL autocommit needs to be turned on.

VTTablet uses connection pools to MySQL. If autocommit was turned off, MySQL will start an implicit transaction (with a point in time snapshot) for each connection and will work very hard at keeping the current view unchanged, which would be counter-productive.

### Safe startup

We recommend to enable `read-only` and `skip-slave-start` at startup.
The first ensures that writes will not be accepted accidentally,
which could cause split brain or alternate futures.
The second ensures that slaves do not connect to the master before
settings like semisync are initialized by vttablet according to
Vitess-specific logic.

### Binary logging

By default, we enable binary logging everywhere (`log-bin`),
including on slaves (`log-slave-updates`).
On *replica* type tablets, this is important to make sure they have the
necessary binlogs in case they are promoted to master.
The slave binlogs are also used to implement Vitess features like
filtered replication (during resharding) and the upcoming update stream
and online schema swap.

### Global Transaction ID (GTID)

Many features of Vitess require a fully GTID-based MySQL replication
topology, including master management, resharding, update stream,
and online schema swap.

For MySQL 5.6+, that means you must use `gtid_mode=ON` on all servers.
We also strongly encourage `enforce_gtid_consistency`.

Similarly, for MariaDB, you should use `gtid_strict_mode` to ensure that
master management operations will fail rather than risk causing data loss
if slaves diverge from the master due to external interference.

### Monitoring

In addition to monitoring the Vitess processes, we recommend to monitor MySQL as well. Here is a list of MySQL metrics you should monitor:

- QPS
- Bytes sent/received
- Replication lag
- Threads running
- Innodb buffer cache hit rate
- CPU, memory and disk usage. For disk, break into bytes read/written, latencies and IOPS.

### Recap

- 2-4 cores
- 100-300GB data size
- Statement based replication (required)
- Semi-sync replication
  - rpl\_semi\_sync\_master\_timeout is huge (essentially never; there's no way to actually specify never)
  - rpl\_semi\_sync\_master\_wait\_no\_slave = 1
  - sync\_binlog=0
  - innodb\_flush\_log\_at\_trx\_commit=2
- STRICT\_TRANS\_TABLES
- auto-commit ON (required)
- Additional parameters as mentioned in above sections.

## Vitess servers

Vitess servers are written in Go. There are a few Vitess-specific knobs that apply to all servers.

### Go version

Go, being a young language, tends to add major improvements over each version.
So, the latest Go version is almost always recommended.
Note that the latest Go version may be higher than the minimum version we require for compiling the binaries (see "Prerequisites" section in the Getting Started guide).

### GOMAXPROCS

You typically don’t have to set this environment variable. The default Go runtime will try to use as much CPU as necessary. However, if you want to force a Go server to not exceed a certain CPU limit, setting GOMAXPROCS to that value will work in most situations.

### GOGC

The default value for this variable is 100. Which means that garbage is collected every time memory doubles from the baseline (100% growth). You typically don’t have to change this value either. However, if you care about tail latency, increasing this value will help you in that area, but at the cost of increased memory usage.

### Logging

Vitess servers write to log files, and they are rotated when they reach a maximum size. It’s recommended that you run at INFO level logging. The information printed in the log files come in handy for troubleshooting. You can limit the disk usage by running cron jobs that periodically purge or archive them.

### gRPC

Vitess uses gRPC for communication between client and Vitess, and between Vitess
servers. By default, Vitess does not use SSL.

Also, even without using SSL, we allow the use of an application-provided
CallerID object. It allows unsecure but easy to use authorization using Table
ACLs.

See the
Transport Security Model document
for more information on how to setup both of these features, and what command
line parameters exist.

## Lock server configuration

Vttablet, vtgate, vtctld need the right command line parameters to find the topo server. First the *topo\_implementation* flag needs to be set to one of *zookeeper* or *etcd*. Then each is configured as follows:

- zookeeper: it is configured using the *ZK\_CLIENT\_CONFIG* environment
  variable, that points at a JSON file that contains the global and local cells
  configurations. For instance, this can be the contents of the file:
  `{"cell1": "server1:port1,server2:port2", "cell2":
  "server1:port1,server2:port2", "global": "server1:port1,server2:port2"}`
- etcd: the *etcd\_global\_addrs* parameter needs to point at the global
  instance. Then inside that global instance, the */vt/cells/* path
  needs to point at each cell instance.

## VTTablet

VTTablet has a large number of command line options. Some important ones will be covered here. In terms of provisioning these are the recommended values

- 2-4 cores (in proportion to MySQL cores)
- 2-4 GB RAM

### Initialization

- Init\_keyspace, init\_shard, init\_tablet\_type: These parameters should be set at startup with the keyspace / shard / tablet type to start the tablet as. Note ‘master’ is not allowed here, instead use ‘replica’, as the tablet when starting will figure out if it is the master (this way, all replica tablets start with the same command line parameters, independently of which one is the master).

### Query server parameters

- **queryserver-config-pool-size**: This value should typically be set to the max number of simultaneous queries you want MySQL to run. This should typically be around 2-3x the number of allocated CPUs. Around 4-16. There is not much harm in going higher with this value, but you may see no additional benefits.
- **queryserver-config-stream-pool-size**: This value is relevant only if you plan to run streaming queries against the database. It’s recommended that you use rdonly instances for such streaming queries. This value depends on how many simultaneous streaming queries you plan to run. Typical values are in the low 100s.
- **queryserver-config-transaction-cap**: This value should be set to how many concurrent transactions you wish to allow. This should be a function of transaction QPS and transaction length. Typical values are in the low 100s.
- **queryserver-config-query-timeout**: This value should be set to the upper limit you’re willing to allow a query to run before it’s deemed too expensive or detrimental to the rest of the system. VTTablet will kill any query that exceeds this timeout. This value is usually around 15-30s.
- **queryserver-config-transaction-timeout**: This value is meant to protect the situation where a client has crashed without completing a transaction. Typical value for this timeout is 30s.
- **queryserver-config-max-result-size**: This parameter prevents the OLTP application from accidentally requesting too many rows. If the result exceeds the specified number of rows, VTTablet returns an error. The default value is 10,000.

### DB config parameters

VTTablet requires multiple user credentials to perform its tasks. Since it's required to run on the same machine as MySQL, it’s most beneficial to use the more efficient unix socket connections.

**app** credentials are for serving app queries:

- **db-config-app-unixsocket**: MySQL socket name to connect to.
- **db-config-app-uname**: App username.
- **db-config-app-pass**: Password for the app username. If you need a more secure way of managing and supplying passwords, VTTablet does allow you to plug into a "password server" that can securely supply and refresh usernames and passwords. Please contact the Vitess team for help if you’d like to write such a custom plugin.
- **db-config-app-charset**: The only supported character set is utf8. Vitess still works with latin1, but it’s getting deprecated.

**dba** credentials will be used for housekeeping work like loading the schema or killing runaway queries:

- **db-config-dba-unixsocket**
- **db-config-dba-uname**
- **db-config-dba-pass**
- **db-config-dba-charset**

**repl** credentials are for managing replication. Since repl connections can be used across machines, you can optionally turn on encryption:

- **db-config-repl-uname**
- **db-config-repl-pass**
- **db-config-repl-charset**
- **db-config-repl-flags**: If you want to enable SSL, this must be set to 2048.
- **db-config-repl-ssl-ca**
- **db-config-repl-ssl-cert**
- **db-config-repl-ssl-key**

**filtered** credentials are for performing resharding:

- **db-config-filtered-unixsocket**
- **db-config-filtered-uname**
- **db-config-filtered-pass**
- **db-config-filtered-charset**

### Monitoring

VTTablet exports a wealth of real-time information about itself. This section will explain the essential ones:

#### /debug/status

This page has a variety of human-readable information about the current VTTablet. You can look at this page to get a general overview of what’s going on. It also has links to various other diagnostic URLs below.

#### /debug/vars

This is the most important source of information for monitoring. There are other URLs below that can be used to further drill down.

##### Queries (as described in /debug/vars section)

Vitess has a structured way of exporting certain performance stats. The most common one is the Histogram structure, which is used by Queries:

```
  "Queries": {
    "Histograms": {
      "PASS_SELECT": {
        "1000000": 1138196,
        "10000000": 1138313,
        "100000000": 1138342,
        "1000000000": 1138342,
        "10000000000": 1138342,
        "500000": 1133195,
        "5000000": 1138277,
        "50000000": 1138342,
        "500000000": 1138342,
        "5000000000": 1138342,
        "Count": 1138342,
        "Time": 387710449887,
        "inf": 1138342
      }
    },
    "TotalCount": 1138342,
    "TotalTime": 387710449887
  },
```

The histograms are broken out into query categories. In the above case, "PASS\_SELECT" is the only category. An entry like `"500000": 1133195` means that `1133195` queries took under `500000` nanoseconds to execute.

Queries.Histograms.PASS\_SELECT.Count is the total count in the PASS\_SELECT category.

Queries.Histograms.PASS\_SELECT.Time is the total time in the PASS\_SELECT category.

Queries.TotalCount is the total count across all categories.

Queries.TotalTime is the total time across all categories.

There are other Histogram variables described below, and they will always have the same structure.

Use this variable to track:

- QPS
- Latency
- Per-category QPS. For replicas, the only category will be PASS\_SELECT, but there will be more for masters.
- Per-category latency
- Per-category tail latency

##### Results

```
  "Results": {
    "0": 0,
    "1": 0,
    "10": 1138326,
    "100": 1138326,
    "1000": 1138342,
    "10000": 1138342,
    "5": 1138326,
    "50": 1138326,
    "500": 1138342,
    "5000": 1138342,
    "Count": 1138342,
    "Total": 1140438,
    "inf": 1138342
  }
```

Results is a simple histogram with no timing info. It gives you a histogram view of the number of rows returned per query.

##### Mysql

Mysql is a histogram variable like Queries, except that it reports MySQL execution times. The categories are "Exec" and “ExecStream”.

In the past, the exec time difference between VTTablet and MySQL used to be substantial. With the newer versions of Go, the VTTablet exec time has been predominantly been equal to the mysql exec time, conn pool wait time and consolidations waits. In other words, this variable has not shown much value recently. However, it’s good to track this variable initially, until it’s determined that there are no other factors causing a big difference between MySQL performance and VTTablet performance.

##### Transactions

Transactions is a histogram variable that tracks transactions. The categories are "Completed" and “Aborted”.

##### Waits

Waits is a histogram variable that tracks various waits in the system. Right now, the only category is "Consolidations". A consolidation happens when one query waits for the results of an identical query already executing, thereby saving the database from performing duplicate work.

This variable used to report connection pool waits, but a refactor moved those variables out into the pool related vars.

##### Errors

```
  "Errors": {
    "Deadlock": 0,
    "Fail": 1,
    "NotInTx": 0,
    "TxPoolFull": 0
  },
```

Errors are reported under different categories. It’s beneficial to track each category separately as it will be more helpful for troubleshooting. Right now, there are four categories. The category list may vary as Vitess evolves.

Plotting errors/query can sometimes be useful for troubleshooting.

VTTablet also exports an InfoErrors variable that tracks inconsequential errors that don’t signify any kind of problem with the system. For example, a dup key on insert is considered normal because apps tend to use that error to instead update an existing row. So, no monitoring is needed for that variable.

##### InternalErrors

```
  "InternalErrors": {
    "HungQuery": 0,
    "Invalidation": 0,
    "MemcacheStats": 0,
    "Mismatch": 0,
    "Panic": 0,
    "Schema": 0,
    "StrayTransactions": 0,
    "Task": 0
  },
```

An internal error is an unexpected situation in code that may possibly point to a bug. Such errors may not cause outages, but even a single error needs be escalated for root cause analysis.

##### Kills

```
  "Kills": {
    "Queries": 2,
    "Transactions": 0
  },
```

Kills reports the queries and transactions killed by VTTablet due to timeout. It’s a very important variable to look at during outages.

##### TransactionPool\*

There are a few variables with the above prefix:

```
  "TransactionPoolAvailable": 300,
  "TransactionPoolCapacity": 300,
  "TransactionPoolIdleTimeout": 600000000000,
  "TransactionPoolMaxCap": 300,
  "TransactionPoolTimeout": 30000000000,
  "TransactionPoolWaitCount": 0,
  "TransactionPoolWaitTime": 0,
```

- WaitCount will give you how often the transaction pool gets full that causes new transactions to wait.
- WaitTime/WaitCount will tell you the average wait time.
- Available is a gauge that tells you the number of available connections in the pool in real-time. Capacity-Available is the number of connections in use. Note that this number could be misleading if the traffic is spiky.

##### Other Pool variables

Just like TransactionPool, there are variables for other pools:

- ConnPool: This is the pool used for read traffic.
- StreamConnPool: This is the pool used for streaming queries.

There are other internal pools used by VTTablet that are not very consequential.

##### TableACLAllowed, TableACLDenied, TableACLPseudoDenied

The above three variables table acl stats broken out by table, plan and user.

##### QueryCacheSize

If the application does not make good use of bind variables, this value would reach the QueryCacheCapacity. If so, inspecting the current query cache will give you a clue about where the misuse is happening.

##### QueryCounts, QueryErrorCounts, QueryRowCounts, QueryTimesNs

These variables are another multi-dimensional view of Queries. They have a lot more data than Queries because they’re broken out into tables as well as plan. This is a priceless source of information when it comes to troubleshooting. If an outage is related to rogue queries, the graphs plotted from these vars will immediately show the table on which such queries are run. After that, a quick look at the detailed query stats will most likely identify the culprit.

##### UserTableQueryCount, UserTableQueryTimesNs, UserTransactionCount, UserTransactionTimesNs

These variables are yet another view of Queries, but broken out by user, table and plan. If you have well-compartmentalized app users, this is another priceless way of identifying a rogue "user app" that could be misbehaving.

##### DataFree, DataLength, IndexLength, TableRows

These variables are updated periodically from information\_schema.tables. They represent statistical information as reported by MySQL about each table. They can be used for planning purposes, or to track unusual changes in table stats.

- DataFree represents data\_free
- DataLength represents data\_length
- IndexLength represents index\_length
- TableRows represents table\_rows

#### /debug/health

This URL prints out a simple "ok" or “not ok” string that can be used to check if the server is healthy. The health check makes sure mysqld connections work, and replication is configured (though not necessarily running) if not master.

#### /queryz, /debug/query\_stats, /debug/query\_plans, /streamqueryz

- /debug/query\_stats is a JSON view of the per-query stats. This information is pulled in real-time from the query cache. The per-table stats in /debug/vars are a roll-up of this information.
- /queryz is a human-readable version of /debug/query\_stats. If a graph shows a table as a possible source of problems, this is the next place to look at to see if a specific query is the root cause.
- /debug/query\_plans is a more static view of the query cache. It just shows how VTTablet will process or rewrite the input query.
- /streamqueryz lists the currently running streaming queries. You have the option to kill any of them from this page.

#### /querylogz, /debug/querylog, /txlogz, /debug/txlog

- /debug/querylog is a never-ending stream of currently executing queries with verbose information about each query. This URL can generate a lot of data because it streams every query processed by VTTablet. The details are as per this function: https://github.com/youtube/vitess/blob/master/go/vt/tabletserver/logstats.go#L202
- /querylogz is a limited human readable version of /debug/querylog. It prints the next 300 queries by default. The limit can be specified with a limit=N parameter on the URL.
- /txlogz is like /querylogz, but for transactions.
- /debug/txlog is the JSON counterpart to /txlogz.

#### /consolidations

This URL has an MRU list of consolidations. This is a way of identifying if multiple clients are spamming the same query to a server.

#### /schemaz, /debug/schema

- /schemaz shows the schema info loaded by VTTablet.
- /debug/schema is the JSON version of /schemaz.

#### /debug/query\_rules

This URL displays the currently active query blacklist rules.

#### /debug/health

This URL prints out a simple "ok" or “not ok” string that can be used to check if the server is healthy.

### Alerting

Alerting is built on top of the variables you monitor. Before setting up alerts, you should get some baseline stats and variance, and then you can build meaningful alerting rules. You can use the following list as a guideline to build your own:

- Query latency among all vttablets
- Per keyspace latency
- Errors/query
- Memory usage
- Unhealthy for too long
- Too many vttablets down
- Health has been flapping
- Transaction pool full error rate
- Any internal error
- Traffic out of balance among replicas
- Qps/core too high

## VTGate

A typical VTGate should be provisioned as follows.

- 2-4 cores
- 2-4 GB RAM

Since VTGate is stateless, you can scale it linearly by just adding more servers as needed. Beyond the recommended values, it’s better to add more VTGates than giving more resources to existing servers, as recommended in the philosophy section.

Load-balancer in front of vtgate to scale up (not covered by Vitess). Stateless, can use the health URL for health check.

### Parameters

- **cells\_to\_watch**: which cell vtgate is in and will monitor tablets from. Cross-cell master access needs multiple cells here.
- **tablet\_types\_to\_wait**: VTGate waits for at least one serving tablet per tablet type specified here during startup, before listening to the serving port. So VTGate does not serve error. It should match the available tablet types VTGate connects to (master, replica, rdonly).
- **discovery\_low\_replication\_lag**: when replication lags of all VTTablet in a particular shard and tablet type are less than or equal the flag (in seconds), VTGate does not filter them by replication lag and uses all to balance traffic.
- **degraded\_threshold (30s)**: a tablet will publish itself as degraded if replication lag exceeds this threshold. This will cause VTGates to choose more up-to-date servers over this one. If all servers are degraded, VTGate resorts to serving from all of them.
- **unhealthy\_threshold (2h)**: a tablet will publish itself as unhealthy if replication lag exceeds this threshold.
- **transaction\_mode (multi)**: `single`: disallow multi-db transactions, `multi`: allow multi-db transactions with best effort commit, `twopc`: allow multi-db transactions with 2pc commit.
- **normalize\_queries (false)**: Turning this flag on will cause vtgate to rewrite queries with bind vars. This is beneficial if the app doesn't itself send normalized queries.

### Monitoring

#### /debug/status

This is the landing page for a VTGate, which can gives you a status on how a particular server is doing. Of particular interest there is the list of tablets this vtgate process is connected to, as this is the list of tablets that can potentially serve queries.

#### /debug/vars

##### VTGateApi

This is the main histogram variable to track for vtgates. It gives you a break up of all queries by command, keyspace, and type.

##### HealthcheckConnections

It shows the number of tablet connections for query/healthcheck per keyspace, shard, and tablet type.

#### /debug/query\_plans

This URL gives you all the query plans for queries going through VTGate.

#### /debug/vschema

This URL shows the vschema as loaded by VTGate.

### Alerting

For VTGate, here’s a list of possible variables to alert on:

- Error rate
- Error/query rate
- Error/query/tablet-type rate
- VTGate serving graph is stale by x minutes (lock server is down)
- Qps/core
- Latency

## External processes

Things that need to be configured:

### Periodic backup configuration

We recommend to take backups regularly e.g. you should set up a cron job for it. See our recommendations at http://vitess.io/user-guide/backup-and-restore.html#backup-frequency.

### Logs archiver/purger

You will need to run some cron jobs to archive or purge log files periodically.

### Orchestrator

Orchestrator is a tool for
managing MySQL replication topologies, including automated failover.
It can detect master failure and initiate a recovery in a matter of seconds.

For the most part, Vitess is agnostic to the actions of Orchestrator,
which operates below Vitess at the MySQL level. That means you can
pretty much
set up Orchestrator
in the normal way, with just a few additions as described below.

For the Kubernetes example, we provide a
sample script
to launch Orchestrator for you with these settings applied.

#### Orchestrator configuration

Orchestrator needs to know some things from the Vitess side,
like the tablet aliases and whether semisync is enforced
(with async fallback disabled).
We pass this information by telling Orchestrator to execute certain
queries that return local metadata from a non-replicated table,
as seen in our sample
orchestrator.conf.json:

```
  "DetectClusterAliasQuery": "SELECT value FROM _vt.local_metadata WHERE name='ClusterAlias'",
  "DetectInstanceAliasQuery": "SELECT value FROM _vt.local_metadata WHERE name='Alias'",
  "DetectPromotionRuleQuery": "SELECT value FROM _vt.local_metadata WHERE name='PromotionRule'",
  "DetectSemiSyncEnforcedQuery": "SELECT @@global.rpl_semi_sync_master_wait_no_slave AND @@global.rpl_semi_sync_master_timeout > 1000000",
```

There is also one thing that Vitess needs to know from Orchestrator,
which is the identity of the master for each shard, if a failover occurs.

From our experience at YouTube, we believe that this signal is too critical
for data integrity to rely on bottom-up detection such as asking each MySQL
if it thinks it's the master. Instead, we rely on Orchestrator to be the
source of truth, and expect it to send a top-down signal to Vitess.

This signal is sent by ensuring the Orchestrator server has access to
`vtctlclient`, which it then uses to send an RPC to vtctld, informing
Vitess of the change in mastership via the
TabletExternallyReparented
command.

```
  "PostMasterFailoverProcesses": [
    "echo 'Recovered from {failureType} on {failureCluster}. Failed: {failedHost}:{failedPort}; Promoted: {successorHost}:{successorPort}' >> /tmp/recovery.log",
    "vtctlclient -server vtctld:15999 TabletExternallyReparented {successorAlias}"
  ],
```

#### VTTablet configuration

Normally, you need to seed Orchestrator by giving it the addresses of
MySQL instances in each shard. If you have lots of shards, this could
be tedious or error-prone.

Luckily, Vitess already knows everything about all the MySQL instances
that comprise your cluster. So we provide a mechanism for tablets to
self-register with the Orchestrator API, configured by the following
vttablet parameters:

- **orc\_api\_url**: Address of Orchestrator's HTTP API (e.g. http://host:port/api/). Leave empty to disable Orchestrator integration.
- **orc\_discover\_interval**: How often (e.g. 60s) to ping Orchestrator's HTTP API endpoint to tell it we exist. 0 means never.

Not only does this relieve you from the initial seeding of addresses into
Orchestrator, it also means new instances will be discovered immediately,
and the topology will automatically repopulate even if Orchestrator's
backing store is wiped out. Note that Orchestrator will forget stale
instances after a configurable timeout.

Contact: vitess@googlegroups.com  **·**  
Announcements  **·**  
© 2017 Vitess powered by Google Inc
